# Study protocol for a multicenter, multinational, observational registry of epidemiology, treatment and outcome of patients with Robin sequence

**DOI:** 10.1186/s13005-023-00364-3

**Published:** 2023-05-20

**Authors:** Anna-Lisa Oechsle, Cornelia Wiechers, Veronique Abadie, Francois Abel, Corstiaan Breugem, Christian F. Poets

**Affiliations:** 1 Interdisciplinary Center for Cleft Palate & Craniofacial Malformations, Department of Neonatology, Tübingen University Hospital, Calwerstr. 7, 72076 Tübingen, Germany; 2 Department of General Pediatrics, Reference Centre for Rare Diseases ’Pierre Robin sequences and congenital sucking-swallowing troubles’, Necker University Hospital, Paris University, &, France; 3 Department of Pediatric Respiratory Medicine, Great Ormond Street Hospital, London, UK; 4 Department of Plastic Reconstructive and Hand Surgery, Emma Children’s Hospital - Location AMC, Amsterdam UMC, University of Amsterdam, Amsterdam, the Netherlands

**Keywords:** Robin sequence, Registry, Database, Rare disease, Outcome

## Abstract

**Background:**

Robin sequence (RS) is a congenital condition characterized by micrognathia, glossoptosis and upper airway obstruction. Diagnosis and treatment are characterized by heterogeneity, resulting in a lack of uniformly collected data.

**Methods:**

We have set up a prospective, observational, multicenter, multinational registry aimed at obtaining routine clinical data from RS patients receiving different treatment approaches and enabling an assessment of outcomes obtained through different therapeutic approaches. Patient enrolment has started in January 2022. Disease characteristics, adverse events and complications depending on the different diagnostic and treatment approaches and their effects on neurocognition, growth, speech development and hearing outcome are evaluated using routine clinical data. In addition to characterizing the patient population and comparing outcomes achieved with different treatment approaches, the registry will evolve to focus on endpoints such as quality of life and long-term developmental status.

**Discussion:**

This registry will provide data on different treatment approaches collected during routine care with diverse framework conditions and will allow assessing diagnostic and therapeutic outcomes of children with RS. These data, urgently demanded by the scientific community, may contribute to refining and personalizing existing therapeutic approaches and increase knowledge about the long-term outcome of children born with this rare condition.

**Trial registration:**

DRKS00025365.

**Supplementary Information:**

The online version contains supplementary material available at 10.1186/s13005-023-00364-3.

## Background

Robin sequence (RS) is a congenital condition characterized by micrognathia, glossoptosis and upper airway obstruction [1, 2]. It is highly variable in phenotype ranging from mild mandibular micrognathia with only occasional obstructive apnea during sleep and undisturbed breathing while awake to life-threatening upper airway obstruction necessitating immediate postnatal intervention to secure the airway and avoid life-threatening hypoxemia. Untreated airway obstruction can lead to intermittent hypoxemia with growth failure, cognitive deficits, and cor pulmonale [3, 4]. The prevalence of RS is estimated at 1:8,500 to 1:14,000 [5–7]. There is evidence suggesting an increased familial risk of the disease. About 80% present with a cleft palate, and half the patients have an isolated diagnosis of RS, while it is associated with other, primarily syndromic diseases in the remaining [8, 9].

There are both non-surgical and surgical treatment options. The former include prone positioning, continuous positive airway pressure (CPAP), use of a nasopharyngeal airway, and orthodontic treatment procedures like special palatal plates and logopedic functional therapy [10–12]. Surgical treatment options include releasing the floor of the mouth, tongue-lip adhesion, mandibular wire traction, mandibular distraction osteogenesis (MDO), or tracheostomy [13–15].

### Utilization of disease registries

As often the case with rare diseases, comparing effectiveness of different treatments using the gold standard, i.e. a randomized controlled trial (RCT) design, is not feasible. Therefore, cohort studies are the only realistic design, and here, registers collecting data from patients treated with different approaches may be helpful, even though the data thus obtained will only be hypothesis-generating. In other rare conditions, registry data have even sufficed instead of RCT to grant market authorization for drugs used to treat them [16, 17].

### Need for a multinational, multicenter clinical registry for RS

Regarding RS, there are few diseases utilizing such diverse therapeutic approaches. Studies on the outcome of children with RS have yet only included one or two of these approaches. Mostly, the parameters and timing of the outcome measures also differed, making it almost impossible to compare study results.

The individual choice of therapy depends on numerous factors and is often determined by the availability of resources, location, as well as experience and expertise of the local interdisciplinary team. Unfortunately, to date, there is no data gathered in a uniform manner enabling a comparison of patients with different therapies and allowing to weigh advantages and disadvantages in a fair and comparable way. In addition, there are hardly any long-term follow-up data on patients with RS. Therefore, little is known about the development and long-term outcome independent of the treatment applied.

### Goals of the registry

We established this registry for patients with RS to systematically gather data in an exhaustive manner on diagnostics, treatment, adverse events and complications, and to increase knowledge about neurocognition, growth, speech development, and hearing outcome in a rigorous manner. The multicentre design enables the collection of data on patients uniformly receiving different treatments.

As stated above, clinical evidence should primarily be based on randomized clinical trials, and comparing treatment approaches based on registry data has limitations [18]. Nonetheless, important evidence can also be obtained using appropriate statistical methods. Without systematically collected data in a registry, specific research questions in rare diseases such as RS cannot be investigated at all.

We will prospectively collect detailed baseline, diagnostic, treatment, and outcome data from several clinical centres specialized in the treatment of RS. The registry aims to describe different diagnostic techniques and the associated measurements under real-life conditions, as well as the impact of different phenotypic varieties on symptoms and treatment. Furthermore, the systematic collection of longitudinal data on characteristics and outcomes enables the detection of factors affecting the latter. For example, we are particularly interested in weight gain and method of feeding in affected infants (i.e., age < 1 year), or inpatient days in the first year of life.

A positive impact of the registry beyond scientific work is the strengthening of international cooperation between RS experts and the facilitation of information exchange on complex cases.

### Effectiveness and safety of different treatment approaches

Another benefit is that a registry not only makes it possible to compare different treatment approaches but can also be used for research into relevant issues related to the respective therapies. Identifying the different diagnostic methods and factors contributing to a good response to each treatment is important as is to confront clinicians with their respective complications and adverse events. This will contribute to a better understanding of disease characteristics and perhaps to a more personalized treatment. In addition to the outlined a priori research questions, it is expected and intended that the availability of registry data will lead to further research questions, unknown at this stage, and provide the opportunity for scientific evaluation.

Since the registry design provides hospitals with a comprehensive database for their own centre, researchers will be further motivated to conduct multiple research in this field. Centres with smaller numbers of cases can also participate, which ultimately increases knowledge about the disease and can improve the quality of care in the long term. Last but not least, information gained from the registry may be helpful in disseminating certain therapeutic and diagnostic procedures in healthcare systems with different jurisdictions and may assist the respective health policymakers in allocating resources.

### Design of the registry

**Fig. 1:**
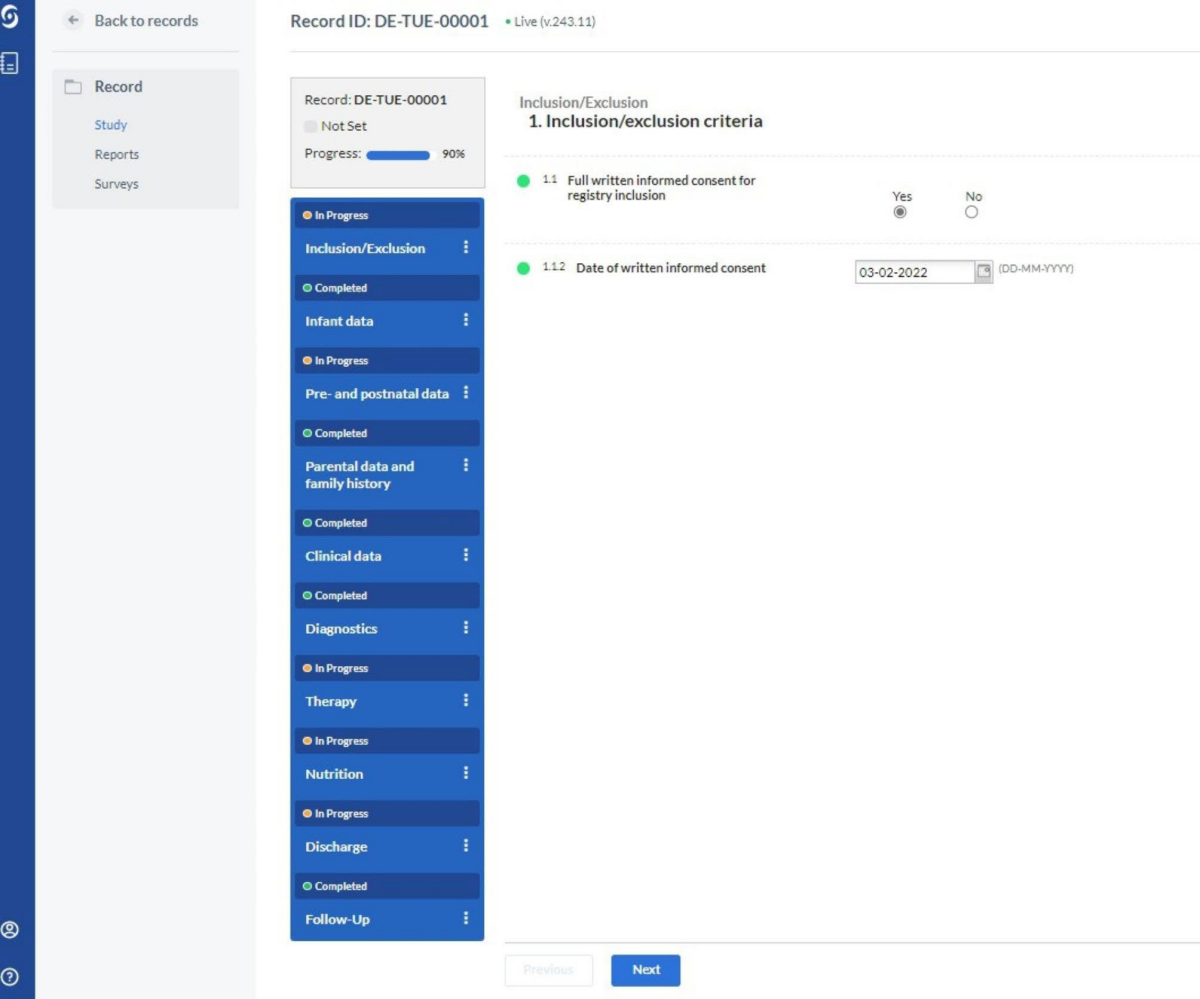
Interface of the registry database for patients with Robin sequence using Castor Electronic Data Capture System (Castor EDC, Ciwit BV, Amsterdam, The Netherlands)

The RS registry is prospective, observational, noninterventional, multicentric and multinational. Data are recorded in an online accessible, secure database using Castor Electronic Data Capture System (Castor EDC, Ciwit BV, Amsterdam, The Netherlands) under license from the university medical centre of Amsterdam (Amsterdam UMC). This user-friendly database enables data entry from every web-connected device (Fig. 1). A user account is required for access, protected by individual passwords and two-factor authentication. Castor applications run on fully managed virtual private servers with providers in the Netherlands. The Castor EDC database complies with the General Data Protection Regulation and is ISO 27,001 certified (Standards for Information Security Assurance). Data is encrypted at rest and in transit.

The database allows the definition of individual user rights and data exporting functions, as well as at least partly extracting data directly from clinical data management systems. Data will be entered pseudonymized directly by physicians and academic staff. The principal investigators of the respective study centres will be required to maintain a secure participation list linking the registry ID number with the person- identifying data. The registry ID is a sequential number that is automatically generated by the system and allows identification of the reporting centre but not the reported patient.

The registry will be conducted under routine clinical conditions, according to the treating physicians. Registry participation does not influence patient management or the relationship between patients and their treating physician. No clinical, instrumental, or laboratory assessments will be performed other than those required for disease management according to each site’s best practice. Follow-up visits will be scheduled according to the standard practice of each centre and the treating physician’s best judgment. In addition to clinical data collection, it is planned to assess topics such as quality of life or neurodevelopment (e.g., developmental milestones) using parental questionnaires.

The idea and design of the register are based on the cooperation of four European centres: Great Ormond Street Hospital for Children NHS in London, the Necker–Enfants Malades Hospital in Paris, the Emma Children’s Hospital in Amsterdam, and the Centre for Cleft Palate and Craniofacial Malformations at the University Children’s Hospital in Tübingen. Each of these four initial centres applies a different treatment modality; all except the UK centre are also members of ERN Cranio. All participating research centres were involved in the item development process to accommodate the diversity of treatment options through item selection. Any hospital treating children with RS can join the registry and participate in the recruitment of patients (readers interested in joining the registry can do so via robinregistry@med.uni-tuebingen.de).

### Case definition and eligibility criteria

All patients diagnosed with RS are eligible for participation in the registry. The diagnosis can be an isolated condition or be part of a syndrome or multiple-anomaly disorder. Patients are eligible irrespective of their treatment. Inclusion is conditional on an informed consent form signed by the child’s legal guardians.

For a long time, definitions for RS differed between studies and centres. However, at an international consensus meeting in Utrecht in 2014, experts agreed on a valid definition also formed the basis for inclusion in this registry. Micrognathia, glossoptosis, and airway obstruction are mandatory diagnostic characteristics [1]. A cleft palate is common in children with RS but not a necessary characteristic of the diagnosis. These three main criteria are queried in the first part of the registry. Generally, borderline cases (cases where the three diagnostic criteria are not clearly identifiable) can also be entered into the registry aiming to facilitate the diagnostic process in unclear cases and to discuss these cases within the expert community.

Since the registry is designed as prospective, patients will be recruited for participation at the earliest possible stage. However, as the time for patients to be admitted to a centre specialized in Robin Sequence varies, we defined the maximum age at registry enrolment as 12 months. The data from the first year of life are essential and should be supplemented retrospectively in case of later recruitment, where possible.

### Data collection procedure and data management

Medical records and data documented in routine care are the primary data sources of this registry, which is designed in two parts. There is a core or minimum data set that must be entered entirely for each participant. The registry is structured into different modules (details of RS phenotype, demographics, pre- and postnatal data, parental data and family history, admission, clinical signs, diagnostics, therapy, adverse events, nutrition, short-term outcome at discharge, and follow-up visits). For each module, there is a second part for which the entry of data is optional. This approach provides flexibility in the volume of data entry for the principal investigators and addresses limited data entry resources. The data entry screen is designed with the aim that data entry should not take longer than 15 min per patient. Each centre regularly can only access its own centre-specific data. From the other centres, only the number of participating patients is visible. The pooling of centre-specific data only takes place within the framework of a research hypothesis and with the consent of all parties involved. This blinding approach is also intended to minimize possible bias in data entry by preventing comparisons being made only at the time of data entry.

The design of the modules will be evaluated regularly. In particular, potential biases in the registry data will be considered. We will re-evaluate the modules with regard to generalizability, selection bias, user bias, confounding, and loss to follow-up. Prior to each data analysis, it is up to the principal investigator to check the correctness and plausibility of the data.

Evaluation and adaptation of the data recording are planned at regular intervals, especially in the initial period of the registry, taking into account the suggestions of participating physicians and scientists. This is a long-term, open-ended registry that is intended to evolve and change with the emerging research questions that the registry can help clarify.

### Dataset

The data set was developed and refined over several years in collaboration with a panel of RS experts. The current data set focuses on the first weeks of primary therapy (see Online Supplement). In particular, an attempt was made to include and standardize routine data already documented for clinical purposes. Nevertheless, centres may be willing to align their observations with the elements of this registry. It is recommended that they modify their diagnostic and follow-up processes without disrupting the course of their usual local treatment.

### Statistical analyses

Appropriate descriptive statistics will be used to describe patient and disease characteristics. Depending on the investigated outcome, characteristics will be presented by the treatment approach. Statistical means such as regression/logistic models will be used in the analysis to evaluate specific effects, taking into account relevant co-variables. Provided that a sufficient number of patients per treatment approach will be enrolled, we plan to conduct a comparison of outcome parameters between the groups using propensity score analysis.

## Ethics and agreements

The ethics boards of the four initial study centres independently approved the registry protocol. The registry complies with all applicable laws and regulations, including Good Clinical Practice and General Data Protection Regulations. Each newly participating centre must submit a positive ethics approval from its local ethics committee for participation. The Steering Group provides study materials, but the responsibility lies with the local principal investigators. Local implementation and administration of local data collection are also the responsibility of the respective study partner. The steering committee is, of course, available to provide support. Participation in the registry also requires the informed consent of the parents/guardians. There are templates provided for the process, but these can and should be adapted to the different policies and regulations in each participating institution and and country. However, the general concept, particularly not to collect person-identifying data at any time, remains unaffected. As only anonymized data are available, linking the data with other sources at the registry level is impossible. The central independent data management is located at the study site of the University Hospital in Tübingen and is responsible for implementing and maintaining the database.

Participating centres must disclose the annual number of cases actually treated and the number of children included in the registry to minimize bias by not including relevant patients. Due to the low expected annual number of cases, it is reasonable to inform all potential study participants and record refusals to participate or reasons for nonenrolment in writing.

The Registry Steering Committee is made up of representatives from the four study centres that launched the registry. The steering group is open to members from any other actively participating center. In case the number of participating centres exceeds the capacity of the steering committee in the future, centres with a larger number of participants will have priority for a seat on the committee. The steering committee plans to meet virtually at least twice a year. All centres participating in the registry are invited to these committee meetings. Centre-specific data are permitted to be used for research purposes independent of the other participating centres. Researchers interested in pooling data from the different participating centres to answer a research question are to submit proposals for specific analysis plans for individual research requests to the Steering Committee for approval before the data are released. In addition to the approval by the Steering Committee, the principal investigator of each centre whose data are to be included in the analysis must also approve the pooling of data and the analysis plan. The steering committee also considers data use requests of researchers not participating in the registry.

## Discussion

This registry for Robin sequence patients is the first registry that includes all patients with RS, irrespective of a particular treatment. Currently, little is known about the outcome of patients with RS, and to date, there is no study comparing outcomes depending on the treatment approach. The longitudinal data collection method of the registry offers the possibility to follow patients over a long time, thus allowing to address previously unstudied research questions. The registry will expand the understanding of diagnosis, disease- and treatment-related conditions, effectiveness of available treatment approaches, and the effect of disease and outcome on family life. As a registry for a rare disease, it fulfills an important role in filling knowledge gaps on RS and strengthening epidemiological and clinical research in this field. In addition, it may help to generate clinical guidelines and thus optimize health care.

The register joins a long line of registers of rare diseases [19, 20], some also covering RS, for example, within the framework of the registers for congenital or craniofacial malformations, clefts or maxillofacial surgery. So far, however, no registry is dedicated to RS and is designed longitudinally, collects outcomes data or records the applied treatment method and its characteristics.

As the registry is designed to collect routine clinical data, and as the clinical setting differs between centres, the quality and completeness of data collection may vary, which might introduce potential bias. Regular inter-centre exchanges on data collection and the development of data entry standards should minimize this limitation.

## Conclusion and outlook

The registry was successfully launched in January 2022. The first patient was included in the same month. As RS is a rare disease, we cannot present any preliminary data at this stage. The first interim analyses are planned after the inclusion of 10 patients per study centre and are expected after about two years of running the registry. The article intents to make the medical community aware that such a registry now exists for this patient group and to encourage them to participate in data collection. Multicentre data from institutions with different treatment schedules and diverse framework conditions will allow assessing diagnostic and therapeutic outcomes of children with RS. The scientific community has urgently demanded such data for years. They will lead centres to rethink their approach, review the quality of their process, and can support the local investigators in requesting funding to establish new processes.

## Electronic supplementary material

Below is the link to the electronic supplementary material.

Supplementary Material 1

## Data Availability

Not applicable. *Competing interests*. The authors declare no conflict of interest.

## References

[1] Breugem CC, Evans KN, Poets CF, Suri S, Picard A, Filip C (2016). Best Practices for the diagnosis and evaluation of infants with Robin sequence: a clinical Consensus Report. JAMA Pediatr.

[2] Evans KN, Sie KC, Hopper RA, Glass RP, Hing AV, Cunningham ML (2011). Robin sequence: from diagnosis to development of an effective management plan. Pediatrics.

[3] Shprintzen RJ (1992). The implications of the diagnosis of Robin sequence. Cleft Palate Craniofac J.

[4] Urschitz MS, von Bodman A, Poets CF (2007). Schlafbezogene Atmungsstörungen im Kindesalter. Monatsschr Kinderheilkd.

[5] Bush PG, Williams AJ (1983). Incidence of the Robin Anomalad (Pierre Robin syndrome). Br J Plast Surg.

[6] Printzlau A, Andersen M (2004). Pierre Robin sequence in Denmark: a retrospective population-based epidemiological study. Cleft Palate Craniofac J.

[7] Wright M, Mehendale F, Urquhart DS (2018). Epidemiology of Robin sequence with cleft palate in the East of Scotland between 2004 and 2013. Pediatr Pulmonol.

[8] Izumi K, Konczal LL, Mitchell AL, Jones MC (2012). Underlying genetic diagnosis of Pierre Robin sequence: retrospective chart review at two children’s hospitals and a systematic literature review. J Pediatr.

[9] Côté A, Fanous A, Almajed A, Lacroix Y (2015). Pierre Robin sequence: review of diagnostic and treatment challenges. Int J Pediatr Otorhinolaryngol.

[10] Downey R, Perkin RM, MacQuarrie J (2000). Nasal continuous positive airway pressure use in children with obstructive sleep apnea younger than 2 years of age. Chest.

[11] Amaddeo A, Abadie V, Chalouhi C, Kadlub N, Frapin A, Lapillonne A (2016). Continuous positive Airway pressure for Upper Airway obstruction in infants with Pierre Robin sequence. Plast Reconstr Surg.

[12] Wiechers C, Arand J, Koos B, Poets CF (2021). Evidence and practical aspects of treatment with the Tübingen palatal plate. Semin Fetal Neonatal Med.

[13] Breugem CC, Logjes RJH, Nolte JW, Flores RL (2021). Advantages and disadvantages of mandibular distraction in Robin sequence. Semin Fetal Neonatal Med.

[14] Baciliero U, Di Spanio Spilimbergo S, Riga M, Padula E (2011). Respiratory distress in Pierre Robin sequence: an experience with mandible traction by wires. Int J Oral Maxillofac Surg.

[15] Papoff P, Guelfi G, Cicchetti R, Caresta E, Cozzi DA, Moretti C (2013). Outcomes after tongue-lip adhesion or mandibular distraction osteogenesis in infants with Pierre Robin sequence and severe airway obstruction. Int J Oral Maxillofac Surg.

[16] Eskola SM, Leufkens HGM, Bate A, de Bruin ML, Gardarsdottir H (2022). Use of Real-World Data and evidence in Drug Development of Medicinal Products centrally authorized in Europe in 2018–2019. Clin Pharmacol Ther.

[17] Flynn R, Plueschke K, Quinten C, Strassmann V, Duijnhoven RG, Gordillo-Marañon M (2022). Marketing authorization applications made to the european Medicines Agency in 2018–2019: what was the contribution of real-world evidence?. Clin Pharmacol Ther.

[18] James S, Daubert J-C, van de Werf F, Commentary (2011). Use of registries to investigate the past and develop the future. BMJ.

[19] Orphanet. Orphanet Report Series: Rare Disease Registries in Europe. 2021. https://www.orpha.net/orphacom/cahiers/docs/GB/Registries.pdf. Accessed 20 May 2022.

[20] Orphanet O. registry/biobank. 2022. https://www.orpha.net/consor/cgi-bin/ResearchTrials_RegistriesMaterials.php?lng=EN&type_list=researchtrials_search_simple_shd&data_id=562&Krankheite(n)/Krankheitsgruppe=Pierre-Robin-Sequenz--isolierte&title=Pierre-Robin-Sequenz,%20isolierte&search=ResearchTrials_RegistriesMaterials_Simple&ChdId=562&ResearchTrials_ResearchTrials_RegistriesMaterials_diseaseGroup=Robin&ResearchTrials_ResearchTrials_RegistriesMaterials_diseaseType=Pat&ResearchTrials_ResearchTrials_RegistriesMaterials_RegCategory=NN&ResearchTrials_ResearchTrials_RegistriesMaterials_country=NN&lng=EN&ResearchTrials_ResearchTrials_RegistriesMaterials_GeographicType=null&ResearchType=Reg. Accessed 20 May 2022.

