# Supplementary material for: Study protocol for a multicenter, multinational, observational registry of epidemiology, treatment and outcome of patients with Robin sequence
Source: Head Face Med. 2023 May 20;19:20. doi: 10.1186/s13005-023-00364-3 (PMC10199645; doi:10.1186/s13005-023-00364-3)
Supplement: Supplementary file 1 — Supplementary Material 1 [file 13005_2023_364_MOESM1_ESM.docx]

| **Form name** | **Variable name** | **Field label** | **C=core,**  **O=optional** | **Option**  **group name** | **Field info** |
| --- | --- | --- | --- | --- | --- |
| Inclusion/exclusion  criteria | consent | Full written informed consent for  registry inclusion | C | yes_no |  |
| Inclusion/exclusion  criteria | exclusion | Reason of exclusion | C | Reason for  exclusion |  |
| Inclusion/exclusion  criteria | exclusion_other | Describe the reason | C |  |  |
| Inclusion/exclusion  criteria | consent_date | Date of written informed consent | C |  |  |
| Details on Robin  sequence | gloss | Glossoptosis? | C | Yes_no_unclear |  |
| Details on Robin  sequence | grad_glossoptosis | Glossoptosis is most applicable to  the patient? | C | grade |  |
| Details on Robin sequence | image1 | Glossoptosis Grade 1: mild glossoptosis | C |  | Mild glossoptosis: the  upper surface of the tongue and the tip of the tongue are visible |
| Details on Robin sequence | image2 | Glossoptosis Grade 2: moderate glossoptosis | C |  | Moderate glossoptosis :  underside of the tongue is visible and in front of the examinator, sublingual ridges are visible but not raised. |
| Details on Robin sequence | image3 | Glossoptosis Grade 3: major glossoptosis | C |  | Major glossoptosis :  tongue is back, only its underside is visible. The tongue may be embedded in the CP. Sublingual ridges are raised. |
| Details on Robin  sequence | mandi | Mandibular micrognathia? | C | Yes_no_unclear |  |
| Details on Robin  sequence | grade_micrognathia | Micrognathia diagnostic | C | grade_micrognathia |  |
| Details on Robin  sequence | Jaw_index | Jaw Index | C |  |  |
| Details on Robin  sequence | overjet | Overjet | C |  |  |
| Details on Robin  sequence | grade_micrognathia_o | Please specify other | C |  |  |
| Details on Robin  sequence | cleft | Cleft palate? | C | Yes_no_unclear |  |
| Details on Robin  sequence | cleft_cat | Please specify cleft palate | C | cleft |  |
| Details on Robin  sequence | uvula | Bifid uvula? | C | Yes_no_unclear |  |
| Details on Robin  sequence | uaw | Upper airway obstruction (UAW) -  clinical and/or diagnostic signs | C | Yes/No |  |
| Details on Robin  sequence | uaw_cat | Diagnosis of UAW based on ... | C | uaw |  |
| Details on Robin  sequence | uaw_o | Please specify other | C |  |  |
| Details on Robin  sequence | uaw_signs | Clinical findings of upper airway  obstruction | C | uaw_signs |  |
| Details on Robin  sequence | uaw_o2 | Please specify other | C |  |  |
| Infant data | gebdat | Date of birth | C |  |  |
| Infant data | sex | Sex | C | sex |  |
| Infant data | gebort | Country of birth | C | Country |  |
| Infant data | admi_date | hospital (any) | C |  |  |
| Infant data | contact_date | Date of first admission to the  specialized center (local hospital) | C |  |  |
| Infant data | transfer | Was there a hospital transfer after  birth (to specialized centers)? | C | Yes/No |  |

| Infant data | dis_date | specialized center) | C |  |  |
| --- | --- | --- | --- | --- | --- |
| Prenatal data | pre_cp | Prenatal diagnosis of cleft palate | C | Yes/No |  |
| Prenatal data | pre_cp_im | by ... | C | Imaging |  |
| Prenatal data | pre_mg | micrognathia | C | Yes/No |  |
| Prenatal data | pre_mg_im | micrognathia by ... | C | Imaging |  |
| Postnatal data | gebort_cat | Place of birth | C | place of birth |  |
| Postnatal data | gebort_cat_o | Specify other place of birth | C |  |  |
| Postnatal data | ga_w | Gestational age at birth | C |  |  |
| Postnatal data | ga_d | Gestational age at birth | C |  |  |
| Postnatal data | multiple | Multiple birth | C | Yes/No |  |
| Postnatal data | Muliple_birth_cat_1 | Please specify how many children  were born? | C |  |  |
| Postnatal data | Muliple_birth_cat_2 | Multiples - Birth order of the child  in question? | C |  |  |
| Postnatal data | gebgewi | Birthweight | C |  |  |
| Postnatal data | gebgr | Length at birth | C |  |  |
| Postnatal data | gebku | Head circumference at birth | C |  |  |
| Postnatal data | gebmode | Mode of delivery | C | Modus |  |
| Postnatal data | agar1 | Apgar- score (1 Min) | C | Apgar |  |
| Postnatal data | apgar5 | Apgar-score (5 Min) | C | Apgar |  |
| Postnatal data | apgar10 | Apgar-score (10 Min) | C | Apgar |  |
| Postnatal data | umbilical_ph | Umbilical cord artery pH-value | C |  |  |
| Additional postnatal data | postnatal_add | Is there any additional postnatal  data available? | O | yes_no |  |
| Additional postnatal data | base_excess | Base excess (in mmol/l) | O |  |  |
| Additional postnatal data | resp | Was respiratory support in  delivery room necessary? | O | Yes/No |  |
| Additional postnatal data | resp_1 | Ex Utero Intrapartum Treatment  (EXIT) Procedure | O | Yes/No |  |
| Additional postnatal data | resp_2 | ET-tube placement and  mechanical ventilation | O | Yes/No |  |
| Additional postnatal data | resp_3 | Chest compression | O | Yes/No |  |
| Additional postnatal data | resp_4 | Bag and mask ventilation | O | Yes/No |  |
| Additional postnatal data | resp_5 | Laryngeal mask airway | O | Yes/No |  |
| Additional postnatal data | resp_6 | Continuous positive airway  pressure (CPAP) | O | Yes/No |  |
| Additional postnatal data | resp_7 | High-flow nasal cannula | O | Yes/No |  |
| Additional postnatal data | resp_8 | Guedel tube or nasopharyngeal  airway | O | Yes/No |  |
| Additional postnatal data | resp_9 | Oxygen administration | O | Yes/No |  |
| Additional postnatal data | resp_11 | Positioning | O | positioning |  |
| Additional postnatal data | resp_10 | Other type of respiratory support  in the delivery room? | O | yes_no |  |
| Additional postnatal data | resp_o | Please describe other(s) | O |  |  |
| Family History | fam_rs | Are there any family members  with Pierre-Robin-sequence? | C | Yes/No |  |
| Family History | fam_rs_cat | Please specify | C | fam |  |
| Family History | fam_rs_o | Please describe other(s) | C |  |  |
| Additional parental data | histoy_add | Is there any additional parental  data available? | O | yes_no |  |
| Additional parental data | fam_cp | Are there any family members  with cleft palate? | O | Yes/No |  |

| Additional parental data | fam_cp_cat | Please specify | O | fam |  |
| --- | --- | --- | --- | --- | --- |
| Additional parental data | fam_cp_o | Please describe other(s) | O |  |  |
| Additional parental data | fam_Stick | Are there any family members  diagnosed with pronounced myopia (nearsightedness), cataract (clouding of the lens), retinal detachment, hearing loss (Arthroophthalmopathy, Stickler | O | Yes/No |  |
| Additional parental data | fam_stick_cat | Please specify | O | fam |  |
| Additional parental data | fam_stick_o | Please describe other(s) | O |  |  |
| Additional parental data | age_ma | Maternal age at delivery (in years) | O |  |  |
| Additional parental data | age_pa | Paternal age at delivery (in years) | O |  |  |
| Additional parental data | edu_ma | Maternal education | O | education |  |
| Additional parental data | edu_pa | Paternal education | O | education |  |
| Additional parental data | fam_sit | Family situation (at admission) | O | family |  |
| Additional parental data | fam_sit_o | Please specify other | O |  |  |
| Additional parental data | gravida | Number of Gravida (incl. this  pregnancy) of the mother | O |  |  |
| Additional parental data | para | Number of Para (incl. this  pregnancy) of the mother | O |  |  |
| Additional parental data | folic | Did the mother use folic in early  pregnancy? | O | Yes/No |  |
| Additional parental data | drug | other medication during  pregnancy? | O | Yes/No |  |
| Additional parental data | drugs_cat | Please specify drugs | O | drugs |  |
| Additional parental data | smoke_num | Number of cigarettes | O |  |  |
| Additional parental data | drugs_o | Please specify other | O |  |  |
| Admission | respi_admi | Respiratory support at admission | C | respi_admi |  |
| Admission | respi_admi_o | Please specify other | C |  |  |
| Admission | gewi_admi | Weight at admission | C |  |  |
| Admission | gr_admi | Length at admission | C |  |  |
| Admission | ku_admi | Head circumference at admission | C |  |  |
| Clinical signs | clinic_add | Do you want to enter data on  clincial signs? | O | yes_no |  |
| Clinical signs | sympt | examination (apart from signs of  RS)? | O | yes_no |  |
| Clinical signs | symp_cat | Please specify further symptoms | O | symptoms |  |
| Clinical signs | symp_o | Please specify other | O |  |  |
| Clinical signs | head_cat | abnormalities | O | head |  |
| Clinical signs | symp_o2 | Please specify other | O |  |  |
| Clinical signs | eyes_cat | Please specify eyes abnormalities | O | eyes_cat |  |
| Clinical signs | symp_o3 | Please specify other | O |  |  |
| Clinical signs | nose_cat | Please specify nose abnormalities | O | nose |  |
| Clinical signs | symp_o4 | Please specify other | O |  |  |
| Clinical signs | mouth_cat | abnormalities | O | mouth |  |
| Clinical signs | symp_o5 | Please specify other | O |  |  |
| Clinical signs | ears_cat | Please specify ears abnormalities | O | ears |  |
| Clinical signs | symp_o6 | Please specify other | O |  |  |

| Clinical signs | chest_cat | Please specify chest abnormalities | O | chest |  |
| --- | --- | --- | --- | --- | --- |
| Clinical signs | symp_o7 | Please specify other | O |  |  |
| Clinical signs | gen_cat | Please specify genitourinary  abnormalities | O | gen_cat |  |
| Clinical signs | symp_o8 | Please specify other | O |  |  |
| Clinical signs | skin_cat | Please specify skin abnormalities | O | skin cat |  |
| Clinical signs | symp_o9 | Please specify other | O |  |  |
| Clinical signs | hair_cat | abnormalities | O | hair |  |
| Clinical signs | symp_o10 | Please specify other | O |  |  |
| Clinical signs | back_cat | Please specify back abnormalities | O | back |  |
| Clinical signs | symp_o11 | Please specify other | O |  |  |
| Clinical signs | limbs_cat | Please specify limb abnormalities | O | limbs |  |
| Clinical signs | symp_o12 | Please specify other | O |  |  |
| Clinical signs | abdom_cat | abnormalities | O | abdom |  |
| Clinical signs | symp_o13 | Please specify other | O |  |  |
| Clinical signs | neuro_cat | abnormalities | O | neuro |  |
| Clinical signs | symp_o14 | Please specify other | O |  |  |
| Genetic tests | gen_synd | Suspected syndromal disease | C | yes_no |  |
| Genetic tests | gen_synd_cat | Please specify | C | synd_2 |  |
| Genetic tests | gen_synd_o | Please specify other (name  ORPHA Code if possible) | C |  |  |
| Genetic tests | gen_add | Is there any additional data on  genetic diagnostics available? | O | yes_no |  |
| Genetic tests | gen_test | Genetic testing? | O | Yes/No |  |
| Genetic tests | gen_test_cat | What kind of genetic testing? | O | gen_test_cat |  |
| Genetic tests | gen_test_o | Please specify other | O |  |  |
| Genetic tests | gen_res | Results of genetic test | O | synd_2 |  |
| Genetic tests | gen_synd_o2 | Please specify other (name  ORPHA Code if possible) | O |  |  |
| Polysomnography | sleep_bf | Sleep study before treatment | C | Yes/No |  |
| Polysomnography | sleep_bf_date | treatment | C |  |  |
| Polysomnography | sleep_cat1 | Measured sleep study parameters | C | sleep |  |
| Polysomnography | ahi | AHI | C |  | Apnea Hypopnea Index: [(Total number of obstructive apneas) + (Total number of mixed apneas) + (Total number of hypopneas associated with >= 3% desat or arousal)] / hours of sleep |
| Polysomnography | moai | MOAI | C |  | Obstructive / Mixed  Apnea Index: [(Total number of  obstructive apneas + Total number of mixed apneas ) / (hours of sleep)] |
| Polysomnography | moahi | MOAHI | C |  | mixed-obstructive apnea-  hypopnea index:  [(sum of obstructive and mixed apneas and all hypopneas ) / (hours of sleep)] |

| Polysomnography | rdi | RDI | C |  | Respiratory Distress  Index:  [(RERAs + Hypopneas  +Total number of all apneas) / (hours of sleep)] |
| --- | --- | --- | --- | --- | --- |
| Polysomnography | di3 | DI3 | C |  | Number of desaturations  <=3% per hour sleep |
| Polysomnography | di90 | DI90 | C |  | Number of desaturations  < 90% per hour sleep |
| Polysomnography | di80 | DI80 | C |  | Number of desaturations  < 80% per hour sleep |
| Polysomnography | av_sao2 | Average SaO2 in sleep | C |  |  |
| Polysomnography | min_saO2 | Minimum SaO2 in sleep | C |  |  |
| Polysomnography | nadir_saO2 | Mean SaO2 nadir | C |  |  |
| Polysomnography | ba_CO2 | Baseline tcpCO2 in sleep | C |  |  |
| Polysomnography | max_CO2 | Maximum tcpCO2 in sleep | C |  |  |
| Polysomnography | CO2_50 | in sleep | C |  |  |
| Polysomnography | sleep_o1 | Please specify other measured  parameter and name the result | C |  |  |
| Polysomnography | sleep_af | after treatment/before first  discharge home? | C | Yes/No |  |
| Polysomnography | sleep_ff_date | treatment | C |  |  |
| Polysomnography | sleep_cat2 | Measured sleep study parameters | C | sleep |  |
| Polysomnography | ahi2 | AHI | C |  | Apnea Hypopnea Index: [(Total number of obstructive apneas) + (Total number of mixed apneas) + (Total number of hypopneas associated with >= 3% desat or arousal)] / hours of sleep |
| Polysomnography | moai2 | MOAI | C |  | Obstructive / Mixed  Apnea Index: [(Total number of  obstructive apneas + Total number of mixed apneas) / (hours of sleep)] |
| Polysomnography | moahi2 | MOAHI | C |  | mixed-obstructive apnea-  hypopnea index:  [(sum of obstructive and mixed apneas and all hypopneas ) / (hours of sleep)] |
| Polysomnography | rdi2 | RDI | C |  | Respiratory Distress  Index:  [(RERAs + Hypopneas  +Total number of all apneas) / (hours of sleep)] |
| Polysomnography | di3_2 | DI3 | C |  | Number of desaturations  <=3% per hour sleep |
| Polysomnography | di90_2 | DI90 | C |  | Number of desaturations  < 90% per hour sleep |
| Polysomnography | di80_2 | DI80 | C |  | Number of desaturations  < 80% per hour sleep |
| Polysomnography | av_sao2_2 | Average SaO2 in sleep | C |  |  |

| Polysomnography | min_saO2_2 | Minimum SaO2 in sleep | C |  |  |
| --- | --- | --- | --- | --- | --- |
| Polysomnography | nadir_saO2_2 | Mean SaO2 nadir | C |  |  |
| Polysomnography | ba_CO2_2 | Baseline tcpCO2 in sleep | C |  |  |
| Polysomnography | max_CO2_2 | Maximum tcpCO2 in sleep | C |  |  |
| Polysomnography | CO2_5_2 | in sleep | C |  |  |
| Polysomnography | slepp_o2 | Please specify other measured  parameter and name the result | C |  |  |
| Additional diagnostic  data | diag_add | Is there any additional diagnostic  data available? | O | yes_no |  |
| Additional diagnostic  data | bloodgas | Blood gas analysis done (1st after  admission to specialized center) | O | Yes/No |  |
| Additional diagnostic  data | blood_pH | pH | O |  |  |
| Additional diagnostic  data | blood_pCO2 | pCO2 | O |  |  |
| Additional diagnostic  data | blood_be | Base excess | O |  |  |
| Additional diagnostic  data | blood_bi | Bicarbonate | O |  |  |
| Additional diagnostic  data | bloodgas_2 | Blood gas analysis done (before  first discharge home) | O | Yes/No |  |
| Additional diagnostic  data | blood_pH_2 | pH | O |  |  |
| Additional diagnostic  data | blood_pCO2_2 | pCO2 | O |  |  |
| Additional diagnostic  data | blood_be_2 | Base excess | O |  |  |
| Additional diagnostic  data | blood_bi_2 | Bicarbonate | O |  |  |
| Additional diagnostic  data | cUS_p | Was a cerebral ultrasound  performed? | O | Yes/No |  |
| Additional diagnostic  data | cUs_cat | Please specify the cUS results | O | US2 |  |
| Additional diagnostic  data | cUS_o | Please specify other pathological  findings | O |  |  |
| Additional diagnostic  data | aUS_p | Was an abdominal ultrasound  performed? | O | Yes/No |  |
| Additional diagnostic  data | aUs_cat | Please specify the aUS results | O | US |  |
| Additional diagnostic  data | aUS_o | Please specify other pathological  findings | O |  |  |
| Additional diagnostic  data | echo | Was an echocardiography  performed? | O | Yes/No |  |
| Additional diagnostic  data | echo_cat | Please specify echocardiography  results | O | echo_cat |  |
| Additional diagnostic  data | echo_o | Please specify other pathological  findings | O |  |  |
| Additional diagnostic  data | bf_2D | 2D photos (side views) before  treatment | O | Yes/No |  |
| Additional diagnostic  data | af_2D | 2D photos (side views) after  treatment | O | Yes/No |  |
| Additional diagnostic  data | mri | Was magnetic resonance imaging  of head/brain performed? | O | Yes/No |  |
| Additional diagnostic  data | mri_date | Date of MRI | O |  |  |
| Additional diagnostic  data | mri_cat | Please specify the MRI results | O | mri_cat |  |
| Additional diagnostic  data | mri_o | Please specify other pathological  findings | O |  |  |
| Additional diagnostic  data | ct_test | Was a CT performed? | O | Yes/No |  |
| Additional diagnostic  data | ct_date | Date of CT | O |  |  |
| Additional diagnostic  data | CT_txt | Please summarise the CT results | O |  |  |

| Additional diagnostic  data | endo | (laryngoscopy/bronchoscopy)  performed? | O | Yes/No |  |
| --- | --- | --- | --- | --- | --- |
| Additional diagnostic  data | endo_cat | Please specify | O | endo_cat |  |
| Additional diagnostic  data | endo_num | Total number done during  hospital stay | O |  |  |
| Additional diagnostic data | endo_res | Please specify the results | O | endo_res | If several were done, refer to the worst results |
| Additional diagnostic  data | endo_o | Please specify other pathological  findings | O |  |  |
| Therapy (part I) | therapy | Was the patient treated with a  specific therapy? | C | Yes/No |  |
| Therapy (part I) | prone | Prone/side Positioning | C | Yes/No |  |
| Therapy (part I) | prone_start | First day of prone/side positioning | C |  |  |
| Therapy (part I) | prone_dis | positioning | C | yes_no |  |
| Therapy (part I) | prone_end | Last day of prone/side positioning | C |  |  |
| Therapy (part I) | cpap | CPAP | C | Yes/No |  |
| Therapy (part I) | cpap_start | First day of CPAP | C |  |  |
| Therapy (part I) | cpapa_dis | Discharge on CPAP | C | yes_no |  |
| Therapy (part I) | cpap_end | Last day of CPAP | C |  |  |
| Therapy (part I) | cpap_days | Total number of days on CPAP | C |  |  |
| Therapy (part I) | cpap_peep | Average Peep for CPAP therapy | C |  |  |
| Therapy (part I) | hfnc | High-Flow nasal cannula | C | Yes/No |  |
| Therapy (part I) | hfnc_start | cannula | C |  |  |
| Therapy (part I) | HFNC_dis | cannula | C | yes_no |  |
| Therapy (part I) | hfnc_end | cannula | C |  |  |
| Therapy (part I) | hfnc_days | Total number of days with High-  Flow nasal cannula | C |  |  |
| Therapy (part I) | hfnc_lmin | cannula | C |  |  |
| Therapy (part I) | intub | Invasive mechanical  Ventilation/Intubation | C | Yes/No |  |
| Therapy (part I) | intub_start | First day of invasive mechanical  ventilation | C |  |  |
| Therapy (part I) | intub_dis | ventilation | C | yes_no |  |
| Therapy (part I) | intub_end | Last day of invasive mechanical  ventilation | C |  |  |
| Therapy (part I) | intub_d | Total number of intubation days  during hospital stay | C |  |  |
| Therapy (part I) | TPP | Tübingen palatal/Pre-epiglottic  baton plate | C | Yes/No |  |
| Therapy (part I) | tpp_start | First day of Tübingen palatal/Pre-  epiglottic baton plate | C |  |  |
| Therapy (part I) | TPP_dis | Discharge with Tübinger  palatal/Pre-epiglottic baton plate | C | yes_no |  |
| Therapy (part I) | tpp_end | Last day of Tübingen palatal/Pre-  epiglottic baton plate | C |  |  |
| Therapy (part I) | TPP_days | Tübinger Palate/Pre-epiglottic  baton plate | C |  |  |
| Therapy (part I) | npa | Nasopharyngeal airway | C | Yes/No |  |
| Therapy (part I) | npa_start | airway | C |  |  |
| Therapy (part I) | npa_dis | airway | C | yes_no |  |
| Therapy (part I) | npa_end | airway | C |  |  |
| Therapy (part I) | npa_days | Total number of days with  Nasopharyngeal airway | C |  |  |
| Therapy (Part II) | tracheo | Tracheotomy | C | Yes/No |  |
| Therapy (Part II) | tracheo_start | Date of tracheotomy | C |  |  |
| Therapy (Part II) | tracheo_dis | Discharge with tracheotomy | C | yes_no |  |

| Therapy (Part II) | tracheo_end | Date of tracheotomy removal | C |  |  |
| --- | --- | --- | --- | --- | --- |
| Therapy (Part II) | tacheo_days | tracheotomy | C |  |  |
| Therapy (Part II) | removal | Tongue-lip adhesion | C | Yes/No |  |
| Therapy (Part II) | removal_op | Date of operation | C |  |  |
| Therapy (Part II) | mandi_tr | Mandibular traction | C | Yes/No |  |
| Therapy (Part II) | mandi_tr_start | First day of Mandibular traction | C |  |  |
| Therapy (Part II) | mandi_tr_dis | Discharge on mandibular traction | C | yes_no |  |
| Therapy (Part II) | mandi_tr_end | Last day of Mandibular traction | C |  |  |
| Therapy (Part II) | mandi_di | osteogenesis | C | Yes/No |  |
| Therapy (Part II) | mandi_di_cat | Please specify | C | distraction |  |
| Therapy (Part II) | mandi_di_start | Date of operation | C |  |  |
| Therapy (Part II) | mandi_di_dis | Discharge with distraction | C | yes_no |  |
| Therapy (Part II) | mandi_di_end | Last day of distraction | C |  |  |
| Therapy (Part II) | floor | Release of the floor of the mouth | C | Yes/No |  |
| Therapy (Part II) | floor_date | Date of operation | C |  |  |
| Therapy (Part II) | therap_o | Was there any other form of  therapy performed? | C | yes_no |  |
| Therapy (Part II) | therap_o_txt | therapy | C |  |  |
| Complementary therapy | ther_com | complementary/paramedical  therapy? | O | yes_no |  |
| Complementary therapy | logo | Logopedic or speech therapy | O | Yes/No |  |
| Complementary therapy | logo_start | therapy | O |  |  |
| Complementary therapy | logo_end | therapy | O |  |  |
| Complementary therapy | physio | Physiotherapy | O | Yes/No |  |
| Complementary therapy | physio_start | Date of start Physiotherapy | O |  |  |
| Complementary therapy | physio_end | Date of end Physiotherapy | O |  |  |
| Complementary therapy | feeding | Special feeding training | O | Yes/No |  |
| Complementary therapy | feeding_start | training | O |  |  |
| Complementary therapy | feeding_end | training | O |  |  |
| Therapy-specific adverse  events (part I) | ther_advers | Do you want to enter data on therapy-specific adverse events? | O | yes_no |  |
| Therapy-specific adverse  events (part I) | skin | Skin irritation on nose or face | O | Yes/No |  |
| Therapy-specific adverse  events (part I) | hypopl | Midface hypoplasia | O | Yes/No |  |
| Therapy-specific adverse  events (part I) | eyes | Sore eyes due to air leak around mask | O | Yes/No |  |
| Therapy-specific adverse  events (part I) | skin2 | Skin irritation at nose or face | O | Yes/No |  |
| Therapy-specific adverse  events (part I) | vocal | Vocal cord granuloma formation | O | Yes/No |  |
| Therapy-specific adverse  events (part I) | steno | Subglottic stenosis | O | Yes/No |  |
| Therapy-specific adverse  events (part I) | bpd | Oxygen requirement >28days or  bronchopulmonary dysplasia (BPD) | O | Yes/No |  |
| Therapy-specific adverse  events (part I) | sedat | Need for sedation or analgesia during ventilation | O | Yes/No |  |
| Therapy-specific adverse  events (part I) | seda_d | How many days? | O |  |  |

| Therapy-specific adverse  events (part I) | skin4 | Skin irritation on the forehead as  a result of fixation the external wires | O | Yes/No |  |
| --- | --- | --- | --- | --- | --- |
| Therapy-specific adverse  events (part I) | marks | Pressure marks on oral mucosa after onset of therapy | O | Yes/No |  |
| Therapy-specific adverse  events (part I) | skin3 | Skin irritation on nose or face | O | Yes/No |  |
| Therapy-specific adverse  events (part I) | obstruc | Acute tube obstruction by nasal secretions | O | Yes/No |  |
| Therapy-specific adverse  events (part I) | steno3 | Nasal stenosis (necessitating intervention) | O | Yes/No |  |
| Therapy-specific adverse  events (part II) | pneumo | Pneumothorax/pneumomediastin um | O | Yes/No |  |
| Therapy-specific adverse  events (part II) | emphys | Subcutaneous emphysema | O | Yes/No |  |
| Therapy-specific adverse  events (part II) | inflam | Inflammation/infection of airway(s) or around tracheostomy | O | Yes/No |  |
| Therapy-specific adverse  events (part II) | bleed | Bleeding | O | Yes/No |  |
| Therapy-specific adverse  events (part II) | decan | Accidental de-cannulation | O | Yes/No |  |
| Therapy-specific adverse  events (part II) | tracheomal | Tracheomalacia/Thinning of the trachea | O | Yes/No |  |
| Therapy-specific adverse  events (part II) | fistula | Tracheoesophageal fistula | O | Yes/No |  |
| Therapy-specific adverse  events (part II) | steno2 | Granulation tissue/subglottic stenosis needing surgical removal | O | Yes/No |  |
| Therapy-specific adverse  events (part II) | feed_issue | Post-operative feeding issues | O | Yes/No |  |
| Therapy-specific adverse  events (part II) | dishi | Dehiscence of adhesion | O | Yes/No |  |
| Therapy-specific adverse  events (part II) | injury | Injury of salivary gland structures | O | Yes/No |  |
| Therapy-specific adverse  events (part II) | speech | Long-term effects on speech production and development | O | Yes/No |  |
| Therapy-specific adverse  events (part II) | speech2 | Speech issues with late release | O | Yes/No |  |
| Therapy-specific adverse  events (part II) | feed_issue2 | Feeding issues | O | Yes/No |  |
| Therapy-specific adverse  events (part II) | wound | Wire-site/wound infection | O | Yes/No |  |
| Therapy-specific adverse  events (part II) | scars | Scars on the chin | O | Yes/No |  |
| Therapy-specific adverse  events (part II) | teeth | Disruption of permanent teeth | O | Yes/No |  |

| Therapy-specific adverse  events (part II) | dislog | Dislodgement or failure of appliance | O | Yes/No |  |
| --- | --- | --- | --- | --- | --- |
| Therapy-specific adverse  events (part II) | conso | Premature consolidation | O | Yes/No |  |
| Therapy-specific adverse  events (part II) | injury2 | Nerve injury (inferior alveolar, marginal mandibular) | O | Yes/No |  |
| Therapy-specific adverse  events (part II) | wound2 | Pin-site/wound infection | O | Yes/No |  |
| Therapy-specific adverse  events (part II) | scar2 | Scarring | O | Yes/No |  |
| Therapy-specific adverse  events (part II) | anky | Ankylosis of mandibular joint | O | Yes/No |  |
| Therapy-specific adverse  events (part II) | ther_advers_o | Other adverse side events? | O | yes_no |  |
| Therapy-specific adverse  events (part II) | ther_advers_o_txt | Please specify others | O |  |  |
| Therapy independant  adverse events | compli | events to report during the  hospital stay? | C | yes_no |  |
| Therapy independant  adverse events | infect | Bacterial infection (need for  antibiotic therapy) | C | Yes/No |  |
| Therapy independant  adverse events | infect_date | Date of bacterial infection | C |  |  |
| Therapy independant  adverse events | sepsis | Sepsis | C | Yes/No |  |
| Therapy independant  adverse events | sepsis_date | Date of bacterial sepsis | C |  |  |
| Therapy independant  adverse events | aspir | Aspiration pneumonia | C | Yes/No |  |
| Therapy independant  adverse events | aspir_date | Date of bacterial aspiration  pneumonia | C |  |  |
| Therapy independant  adverse events | compli_o | Any other complications to  report? | C | yes_no |  |
| Therapy independant  adverse events | compli_o_txt | Please describe | C |  |  |
| Medication | medi | Medication? | O | yes_no |  |
| Medication | antibio | Antibiotics | O | Yes/No |  |
| Medication | antibio_start | Start date of antibiotics | O |  |  |
| Medication | antibio_ongoing | Ongoing antibiotics at discharge? | O | Yes/No |  |
| Medication | antibio_end | End date of antibiotics | O |  |  |
| Medication | anticon | Anticonvulsant drugs | O | Yes/No |  |
| Medication | anticon_txt | Generic name of the drug | O |  |  |
| Medication | anticon_start | Start date of anticonvulsant drugs | O |  |  |
| Medication | anticon_ongoing | Ongoing anticonvulsant drugs at  discharge? | O | Yes/No |  |
| Medication | anticon_end | End date of anticonvulsant drugs | O |  |  |
| Medication | antiref | Anti-reflux therapy | O | Yes/No |  |
| Medication | antiref_cat | Please specify | O | antireflux |  |
| Medication | antiref_o | Please specify other | O |  |  |
| Medication | antiref_start | Start date of anti-reflux therapy | O |  |  |
| Medication | antiref_ongoing | discharge? | O | Yes/No |  |
| Medication | antiref_end | End date of anti-reflux therapy | O |  |  |
| Medication | seda | Sedation | O | Yes/No |  |
| Medication | seda_o | Please report name(s) of the  sedative drug(s) | O |  |  |

| Medication | seda_start | Start date of sedation | O |  |  |
| --- | --- | --- | --- | --- | --- |
| Medication | seda_ongoing | Ongoing sedation at discharge? | O | Yes/No |  |
| Medication | seda_end | Date of end of sedation | O |  |  |
| Medication | medi_o | Other medication | O | yes_no |  |
| Medication | medi_o_txt | Please report the name of the  other(s) drug(s) | O |  |  |
| Nutrition | food_mode2 | How was the child fed during the  hospital stay | C | feed_mode | Multiple answers possible |
| Nutrition | food_mode_o3 | Please specify other | C |  |  |
| Nutrition | full_oral_date | Date of fully orally feed | C |  |  |
| Nutrition | food_mode | Oral feeding mode: how is the  child fed (predominantly)? | C | food_mode |  |
| Nutrition | food_mode_o | Please specify other | C |  |  |
| Nutrition | breat_training | Has breastfeeding training been  carried out on the child? | C | Yes/No |  |
| Nutrition | breast_50 | breastfed? | C | Yes/No |  |
| Nutrition | tube_dis | Discharge with nasogastric tube? | C | Yes/No |  |
| Nutrition | tube_remove_date | Date of removal | C |  |  |
| Nutrition | stoma_date1 | Date of surgery | C |  |  |
| Nutrition | gastro_dis | Discharge with gastrostomy? | C | Yes/No |  |
| Nutrition | gastro_remove_date | Date of removal | C |  |  |
| Additional nutrition data | food_add | Is there any additional data on  nutrition available? | O | yes_no |  |
| Additional nutrition data | fodd_diff | Where there any feeding  difficulties (before treatment)? | O | Yes/No |  |
| Additional nutrition data | fodd_diff2 | Prolonged feeding times (e.g. >  30minutes with 1 bottle feed) | O | Yes/No |  |
| Additional nutrition data | fodd_diff3 | Worsening of airway obstruction  during oral feeding | O | Yes/No |  |
| Additional nutrition data | fodd_diff4 | Signs of Aspiration (eg. coughing  while drinking, radiological signs, recurrent pneumonia) | O | Yes/No |  |
| Additional nutrition data | fodd_diff5 | Weak/Poor Sucking | O | Yes/No |  |
| Additional nutrition data | fodd_diff6 | clinical signs like increased  salivation) | O | Yes/No |  |
| Short term outcome | dis_r | Reason for discharge | C | discharge |  |
| Short term outcome | death_date | Date of death | C |  |  |
| Short term outcome | dis_final_date | Date of final discharge home (end  of inpatient treatment) | C |  |  |
| Short term outcome | dis_final_date2 | Date of final discharge home  (after rehabilitation) | C |  |  |
| Short term outcome | dis_final_date3 | Date of final discharge home | C |  |  |
| Short term outcome | dis_r_o | Please specify other | C |  |  |
| Short term outcome | gewi_dis | Weight at discharge | C |  |  |
| Short term outcome | gr_dis | Length at discharge | C |  |  |
| Short term outcome | ku_dis | Head circumference at discharge | C |  |  |
| Short term outcome | dis_o2 | requirement | C | Yes/No |  |
| Short term outcome | dis_monitor | monitoring? | C | Yes/No |  |
| Short term outcome | food | Type of food at discharge | C | food | Multiple answers possible |
| Short term outcome | food_o | Please specify other | C |  |  |
| Follow-up Visits | fu_2 | measurements | O |  |  |
| Follow-up Visits | fu_4 | Report a follow-up sleep study | O |  |  |
| Follow-up Visits | fu_3 | Report cleft palate operations | O |  |  |
| Follow-up Visits | fu_5 | Report neurological examination | O |  |  |

| Follow-up Visits | fu_6 | Report death or an apparent life-  threatening event (ALTE) | O |  |  |
| --- | --- | --- | --- | --- | --- |
| Follow-up Visits | fu_7 | Report inpatient stays during 1st  year of life | O |  |  |
